# SLAM-family receptors come of age as a potential molecular target in cancer immunotherapy

**DOI:** 10.3389/fimmu.2023.1174138

**Published:** 2023-05-11

**Authors:** Pooya Farhangnia, Shamim Mollazadeh Ghomi, Shabnam Mollazadehghomi, Hamid Nickho, Mahzad Akbarpour, Ali-Akbar Delbandi

**Affiliations:** ^1^ Immunology Research Center, Institute of Immunology and Infectious Disease, Iran University of Medical Sciences, Tehran, Iran; ^2^ Department of Immunology, School of Medicine, Iran University of Medical Sciences, Tehran, Iran; ^3^ Immunology Board for Transplantation and Cell-Based Therapeutics (ImmunoTACT), Universal Scientific Education and Research Network (USERN), Tehran, Iran; ^4^ Advanced Cellular Therapeutics Facility (ACTF), Hematopoietic Cellular Therapy Program, Section of Hematology & Oncology, Department of Medicine, University of Chicago Medical Center, Chicago, IL, United States

**Keywords:** SLAM, cancer immunotherapy, elotuzumab, CD244 (2B4), CD150, CD84, CD229, SLAMF7/CS1

## Abstract

The signaling lymphocytic activation molecule (SLAM) family receptors were discovered in immune cells for the first time. The SLAM-family receptors are a significant player in cytotoxicity, humoral immune responses, autoimmune diseases, lymphocyte development, cell survival, and cell adhesion. There is growing evidence that SLAM-family receptors have been involved in cancer progression and heralded as a novel immune checkpoint on T cells. Previous studies have reported the role of SLAMs in tumor immunity in various cancers, including chronic lymphocytic leukemia, lymphoma, multiple myeloma, acute myeloid leukemia, hepatocellular carcinoma, head and neck squamous cell carcinoma, pancreas, lung, and melanoma. Evidence has deciphered that the SLAM-family receptors may be targeted for cancer immunotherapy. However, our understanding in this regard is not complete. This review will discuss the role of SLAM-family receptors in cancer immunotherapy. It will also provide an update on recent advances in SLAM-based targeted immunotherapies.

## 1 Introduction

Cancer is the second most common cause of death before the age of 70, according to data from the World Health Organization (WHO). There are several cancer therapies available. They are selected for each patient depending on the kind of cancer and the stage of the disease at diagnosis. Chemotherapy, radiation therapy, stem cell transplantation, surgery, and immunotherapy are some treatments (1).

Cancer immunotherapy approaches may induce a considerable anti-tumor response. These may be achieved *via* various strategies, including immune checkpoint inhibitors (ICIs) and adoptive cellular therapies. Clinical trials treating patients with various cancers between 2010 and the present have shown impressive results employing various immunotherapeutic approaches. Immunotherapy-induced anti-tumor responses can persist for a considerable time after the treatment has ceased, unlike responses from therapies that target the tumor directly (1). Programmed cell death-1 (PD-1), cytotoxic T-lymphocyte-associated protein 4 (CTLA-4), and programmed cell death ligand 1 (PD-L1) are examples of inhibitory immune checkpoint molecules (ICMs) that suppress immune responses and allow tumor immune escape. These ICMs are expressed by immune cells and tumor cells. Several ICIs, including ipilimumab, which targets CTLA-4, and nivolumab, which targets PD-1, have been authorized by the U.S. Food and Drug Administration (FDA) in the past ten years to treat a variety of cancers (2).

Signaling lymphocytic activation molecule (SLAM) family receptors were discovered in immune cells for the first time. In humans and mice, the genes that code for SLAM-family receptors are clustered on chromosome 1 in a 400 kilobase (kb) cluster (3). Recently, some members of this family have been identified as inhibitory immune checkpoints (4, 5). These receptors engage both activating and inhibitory SH2 domain-containing proteins *via* their immunoreceptor tyrosine-based switch motifs (ITSMs), which are found on both the activating and inhibitory SH2 domain-containing proteins. Evidence indicates that this family’s members are engaged in various physiological and pathological processes, including the regulation of immunological responses and the route of viral entry (6). The discovery of a genetic abnormality responsible for X-linked lymphoproliferative (XLP) disease highlights the functional relevance of SLAM molecules in immune response (7)—the XLP gene mutation codes for SLAM-associated protein (SAP) or SH2D1A, a small adapter-like protein. SAP comprises the Src homology 2 (SH2) domain and binds to tyrosines in the intracellular domain of SLAM-related receptors with great specificity and affinity (8). SLAM receptors in humans form a receptor complex when they bind with SAP and Ewing’s sarcoma-activated transcript 2 (EAT-2). Also, the EAT-2-related transducer (ERT; SH2D1W) is a member of the SAP family (9, 10).

Previous studies have reported the role of SLAMs in tumor immunity in a variety of cancers, including chronic lymphocytic leukemia (CLL) (11), lymphoma (12), multiple myeloma (MM) (13), acute myeloid leukemia (AML) (14), hepatocellular carcinoma (HCC) (15), head and neck squamous cell carcinoma (HNSCC) (16), pancreas (17), lung (17), and melanoma (18). Anti-CTLA-4 and anti-PD-1 mAbs have been incredibly effective. However, not all patients benefit from them. Therefore, the discovery of novel ICMs would facilitate the development of novel targeted immunotherapies, new drugs, enhanced therapeutic response, and improved patients survival. Thus, this review will focus on the current knowledge of the role of the SLAM family of receptors in regulating immune functions during tumor progression and recent findings describing how these molecules can be exploited as a novel target in cancer immunotherapy. As described in this review, several anti-SLAM mAbs are now under exploration in various cancer clinical trials.

## 2 SLAM-family receptors: expression, function, and signaling

**Figure 1:**
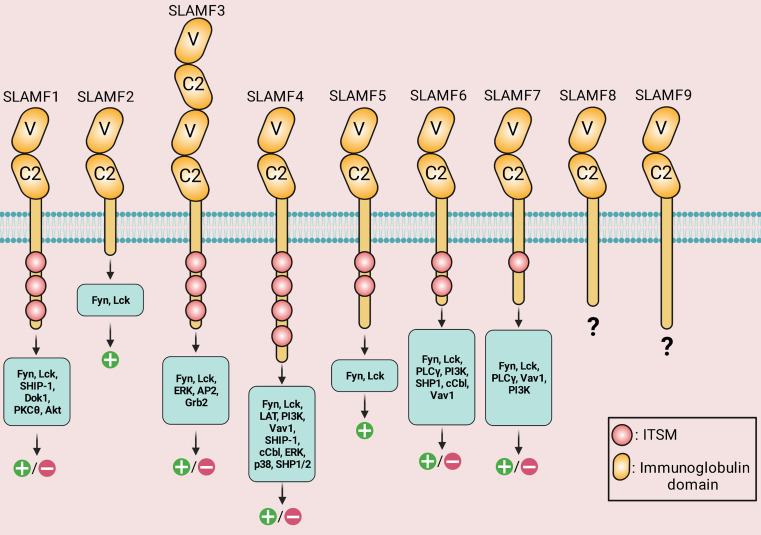
Structure, downstream signaling molecules, and the known signaling outcomes of signaling lymphocytic activation molecule (SLAM)-family receptors. IgV (V)- and IgC2 (C2)-like domains are found in the extracellular areas. *Via* the IgV-like domain, the receptors attach to self-ligands with great affinity. The cytoplasmic tail of every family member, except for SLAMF2, SLAMF8, and SLAMF9, has an immunoreceptor tyrosine-based switch motif (ITSM). SLAMF4 has the highest number of ITSM motifs. SLAMF3 has four Ig-like domains in its extracellular domain. Positive symbols and negative symbols indicate activatory outcome and inhibitory outcome, respectively.

**Figure 2:**
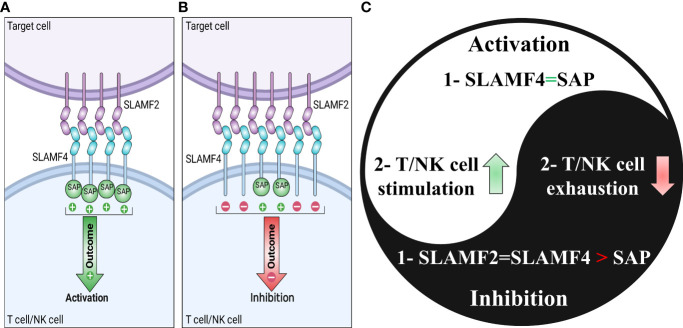
Signaling lymphocytic activation molecule-family receptor (SLAMF) 4-SLAMF2 interaction and dichotomous function of SLAMF4. SLAMF4 can affect T cells and natural killer (NK) cells in an activating or inhibiting manner. In the presence of adequate quantities of the adaptor protein SAP on the target cell, the SLAMF4-SLAMF2 interaction promotes NK or T cell activation **(A)**. However, it results in an inhibitory signal in the absence of SAP on the target cell. **(C)** The Yin-yang of the SLAMF4 dual functions summarizes the parts of **(A, B)**.

The SLAM family of receptors is a group of type I transmembrane receptors that includes SLAMF1 (CD150; SLAM), SLAMF2 (CD48), SLAMF3 (Ly-9; CD229), SLAMF4 (CD244; 2B4), SLAMF5 (CD84), SLAMF6 (Ly108; NTB-A; CD352), SLAMF7 (CRACC; CS1; CD319), SLAMF8 (BLAME; CD353), and SLAMF9 (CD84-H1; SF2001; CD2F10) (**Table 1**) (6). All molecules of the SLAM family are members of the Immunoglobulin (Ig) superfamily. Each SLAM molecule has an extracellular segment consisting of two Ig-like domains (V-like variable and C2-like constant), a transmembrane segment, and a cytoplasmic tail bearing multiple tyrosine-based motifs TxYxxI/V (ITSM), (T is threonine, I is isoleucine, V is valine and X is any amino acid). The one exception to this structural organization is SLAMF3, which has four Ig-like domains in its extracellular domain. Additionally, SLAMF2, SLAMF8, and SLAMF9 lacks ITSM domains (**Figure 1**) (6). In immune cells that contain SLAM-family receptors containing ITSMs, SLAM/SAP and SLAM/EAT-2 complexes interact directly with Src family kinase Fyn and phospholipase Cγ, respectively, and stimulate an activating signal within these immune cells (76). On the one hand, groundbreaking studies revealed that SLAM molecules acted as activating receptors in immune cells that expressed adaptors belonging to the SAP family. However, those same receptors acted as inhibiting receptors in cells devoid of these adaptors. On the other hand, it has been revealed that SLAM-family receptors, particularly SLAMF4, might be either inhibitory or activating in cells carrying SAP family adaptors, depending on the circumstances (**Figure 2**) (7).

**Table 1:** SLAM-family receptors.

SLAM Receptor	Name	Ligand_(s)_	Expression Pattern	Number of ITSM Motif	Binding to SAP	Binding to EAT-2 (ERT)	Adaptor Proteins in Signaling	References
SLAMF1	SLAM, CD150, IPO-3	1- SLAMF1 2- Measles virus hemagglutinin 3- OmpC and OmpE from gram-negative bacteria	T, B, DC, MQ, Plt, HSC, thymocyte, TFH, NK	Human: 2 Mouse: 2	+	+	Fyn, Lck, SHIP-1, Dok1, PKCθ, Akt	(19–36)
SLAMF2	CD48, BLAST-1	1- CD2 2- SLAMF4 3- Type-1 fimbriae	NK, B, T CD8^+^, γδ T, basophil, Eos, MC, Neu, monocyte	None	None	None	Fyn, Lck	(27, 37–39)
SLAMF3	CD229, Ly-9	SLAMF3	DN and DP thymocyte, T, TFH, B, DC, MQ, NK	Human: 2 Mouse: 2	+	+	Fyn, Lck, ERK, AP2, Grb2	(40–45)
SLAMF4	2B4, CD244	SLAMF2	T CD8^+^, γδ T, NK, MQ, basophil, MC, Eos	Human: 4 Mouse: 4	+	+	Fyn, Lck, LAT, PI3K, Vav1, SHIP1, cCbl, ERK, p38, SHP1, SHP2	(27, 46–55)
SLAMF5	CD84	SLAMF5	SP thymocyte, T, TFH, B, MQ, DC, Plt, MC, basophil, Eos, CD34^+^ HSC	Human: 2 Mouse: 2	+	+	Fyn, Lck	(22, 26, 34, 45, 50, 56–60)
SLAMF6	Ly108, NTB-A, CD352	SLAMF6	DN and SP thymocyte, T, B, DC, NK, Neu, Eos	Human: 2 Mouse: 2	+	+	Fyn, Lck, PLCγ, PI3K, SHP1, cCbl, Vav1	(20, 22, 61–64)
SLAMF7	CRACC, CS1, CD319	SLAMF7	NK,B,T,MQ,DC,PC	Human: 1 Mouse: 1	+	+	Fyn, Lck, PLCγ, Vav1, PI3K	(65–68)
SLAMF8	BLAME, CD353	Not identified	Neu,MQ,monocyte,DC,T,NK	None	Not identified	Not identified	Not identified	(69–71)
SLAMF9	CD84-H1, SF2001, CD2F10	Not identified	pDC, TAM, MQ, peritoneal B1 cell, myeloid cell	None	Not identified	Not identified	Not identified	(72–75)

SLAM, Signaling lymphocytic activation molecule; T, T cell; B, B cell; DC, Dendritic cell; MQ, Macrophage; TFH, T follicular helper cell; Plt, Platelet; Eos, Eosinophil; NK, Natural killer cell; Neu, Neutrophil; TAM, Tumor associated macrophage; pDC, Plasmacytoid dendritic cell; PC, Plasma cell; MC, Mast cell; SP, Single positive; DN, Double negative; DP, Double positive; HSC, Hematopietic stem cell; SAP, Signaling lymphocytic activation molecule-associated protein; EAT-2, Ewing’s sarcoma-associated transcript-2; ERT, EAT-2–related transducer.

Glycosylphosphatidylinositol (GPI) anchor is used by SLAMF2 (6, 7, 77). Unlike most receptors expressed on immune cells, SLAM molecules are routinely self-ligands and have homotypic cell-cell interaction. However, there is an exception. For instance, SLAMF2 interacts with SLAMF4, a heterophilic interaction (78). Overall, the SLAM family of receptors can be stimulated *via* homotypic or heterotypic cell-cell interactions.

### 2.1 SLAMF1

The glycoprotein SLAMF1 or CD150 is found in T, B, natural killer (NK), and dendritic cells (DCs; **Table 1**). SLAMF1 extracellular domain is a measles virus receptor, and SLAMF1 functions as a co-stimulator on T and B cells. Although type 1 T helper (Th1) cells have significant levels of SLAMF1, Th2 cells have only trace quantities (19). SLAMF1 membrane proteins are found in plasma cells (79). Findings have indicated that normal B cells and B lymphoblastoid cell lines express SLAMF1 at equivalent mRNA and protein levels (80). SLAMF1 is involved in T cell and B cell activation and differentiation, germinal center formation, and antibody production (81). It is also expressed on thymocytes, where it is involved in thymocyte development and positive selection (20, 82).

In normal immune cells, SLAMF1 is known to recruit and activate intracellular signaling molecules, such as SH2-containing tyrosine phosphatase 2 (SHP-2), phosphatidylinositol 3-kinase (PI3K), and the mitogen-activated protein kinases (MAPKs) p38, upon binding to its ligands (77, 83). While, in tumor cells, SLAMF1 has been found to be up-regulated, and it regulates cell growth and survival (84). SLAMF1 is known to mediate the survival and growth of tumor cells by activating a PI3K/Akt/mTOR signaling pathway, which is involved in regulating cell metabolism, proliferation, and survival (85–88).

### 2.2 SLAMF2 and SLAMF4

SLAMF2 (CD48) is a glycoprotein related to glycophosphatidylinositol expressed on the surface of immune cells such as mature lymphocytes and monocytes and functions as a ligand for SLAMF4 (**Table 1**). SLAMF2 is abundantly expressed on T cells, B cells, and plasma cells and is expressed at a lesser level on monocytes, neutrophils, and CD34^+^ cells but not on platelets or red blood cells (13, 78). SLAMF2 is expressed by human innate lymphoid cell (ILC) progenitors, which regulate the differentiation of these cells (89).

SLAMF4, also known as 2B4 or CD244, is a member of the immunoglobulin (Ig) superfamily, which belongs to the CD2 subgroup. SLAMF4 is expressed on the cell surfaces of NK cells, T cells, monocytes, eosinophils, myeloid-derived suppressor cells (MDSCs), and DCs (**Table 1**). SLAMF4 interacts with the ligand SLAMF2 on target cells to regulate immune activity and transmits stimulatory or inhibitory signals (**Figure 2**) (90). In the intestinal mucosa, SLAMF4 regulates the fine balance between tolerance maintenance and immune surveillance (91). SLAMF4 is a negative regulator of cytotoxic intraepithelial CD8^+^ T cell expansion in the small intestine, maintaining homeostasis (92).

In normal immune cells, the interaction between SLAMF2 and SLAMF4 involves the recruitment of various signaling molecules, including SAP and Src-family kinases such as Fyn and Lyn. Once activated, SLAMF4 signaling leads to downstream activation of MAPK and PI3K/Akt signaling pathways, which are essential for T cell activation and proliferation (76). In hematopoietic tumors, SLAMF4 hinders signals activating mTOR and Syk through the recruitment of SHP-2 (4). Furthermore, the interaction between SLAMF2 and SLAMF4 involves the recruitment of kinases such as LCK. Once activated, SLAMF2 signaling leads to downstream activation of MAPK and PI3K/Akt signaling pathways, which are essential for T cell activation and proliferation (76). Furthermore, the presence of SLAMF2 in DCs stimulates the activation of NK cells that express SLAMF4 through the activation of PLCγ, the induction of $\text{C}{\text{a}}_{2}^{+}$ flux and the activation of ERK mediated by SAP or EAT-2 (93). If SAP and EAT-2 are not present, the interaction between SLAMF2 and SLAMF4 stimulates a signal that restrains NK cells with the help of proteins SHP1, SHP2, and SHIP-1 (7, 94–97). When the protein SAP is present in sufficient amounts, the interaction between SLAMF2 and SLAMF4 can help activate T cells or NK cells. As stated, the interaction between SLAMF2 and SLAMF4 has both co-stimulatory and co-inhibitory roles in immune cells. However, the specific function depends on whether SAP or EAT-2 expression is present (**Figure 2**) (76).

### 2.3 SLAMF3

SLAMF3 (also known as Ly-9 or CD229) is expressed in T and B cells, NK cells, and thymocytes (**Table 1**) (40). SLAMF3 plays a role in diabetes in such a way that palmitic acid activates SLAMF3 on human T cells through the STAT5-PI3K/Akt pathway, exhibiting the chronic inflammatory characteristics of type 2 diabetes (98). The lack of the SLAMF3 revealed that this receptor negatively impacts B cell homeostasis by modifying the activation thresholds of B cell subsets in mice (99).

The immune synapse is formed by SLAM receptors clustering, including SLAMF1/3/5/6, which brings about the activation of Fyn through SAP binding to the cytoplasmic tails. This leads to the activation of PKCθ and Bcl-10, which in turn induces nuclear factor kappa B (NF-κB) activation and the production of Th2 cytokines (77, 100). Consequently, this plays a role in developing natural killer T (NKT) cells and CD4^+^ T cells. The cytoplasmic tails of SLAM receptors containing phosphorylated tyrosines associate with SHIP, docking protein 1 (DOK1), DOK2, and RAS-GAP, which can potentially take part in these processes (77). In hematopoietic tumors, SLAMF3 is categorized as a “don’t eat me” receptor present on macrophages. Its primary function is to hinder or inhibit “eat me” signals that activate mTOR and Syk, which are typically initiated by LRP1, through SHP-2 (4). In MM, SLAMF3 directly binds to the SHP-2 and growth factor receptor bound 2 (GRB2), which eventually activates downstream pathways, including MAPK/ERK signals (101).

### 2.4 SLAMF5

SLAMF5 (CD84) is expressed on CD34^+^ hematopoietic progenitor cells, monocytes, macrophages, and B/T lymphocytes (**Table 1**). SLAMF5 expression reduces the proliferative ability of CD34^+^ cells, indicating that it is a marker for hematopoietic progenitor cells (102, 103). SLAMF5 is a negative moderator required to maintain and function IL-10^+^ regulatory B cells (104). In monocyte-derived DCs, SLAMF5 regulates autophagy and stabilizes interferon regulatory factor 8 (IRF8) (105). SLAMF5 acts as a negative regulator of the development of innate CD8^+^ T cells (106) and humoral immune response (107).

SLAMF5 through SAP binding helps regulate the intensity of T cell receptor (TCR) signals in primary T cell responses, which contributes to p73-mediated apoptosis. This process involves the recruitment of Fyn, which fine-tunes the TCR signal strength. Activated Fyn can impede Itch, a ubiquitin ligase, thereby preventing the degradation of p73 (77, 108). However, in tumor cells, SLAMF5-mediated signaling promotes cell survival (109). Thus, further research is needed to shed light on this discrepancy of SLAMF5 signaling in different contexts.

### 2.5 SLAMF6

SLAMF6 (sometimes referred to as Ly108, NTB-A, or CD352) is an immunoglobulin superfamily homophilic cell-surface receptor and a type I transmembrane protein. SLAMF6 has two immunoglobin (Ig)-like extracellular domains and two ITSMs (61, 62, 110). The SLAMF6 receptor is found on the surface of a wide range of hematopoietic cells, including T cells, B cells, and NK cells (expression limited to humans; **Table 1**), and interactions between these cell types allow for a variety of immunomodulatory functions, including adhesion, innate T-lymphocyte development, neutrophil function, and NK and CD8^+^ T cell-mediated cytotoxicity (20, 111, 112).

On the one hand, in the T cell activation phase, SLAMF6 upon binding to its ligand can recruit Fyn, which in turn impedes the Itch molecule, resulting in p73-mediated apoptosis. On the other hand, when T cell enters their re-activation phase, SLAMF6 could prompt apoptosis. This apoptosis-inducing effect occurs *via* a mechanism that is dependent on SAP, but not on Fyn. According to this mechanism, SAP helps remove SHP1 from the cytoplasmic tail of SLAMF6. This, in turn, leads to stronger signaling from TCR, which eventually results in the expression of genes such as FasL and Bim that mediate the process of restimulation-induced cell death (RICD) (77, 108). Furthermore, as mentioned earlier, SLAMF6 can activate PKCθ and Bcl-10, which in turn induces NF-κB activation and the production of Th2 cytokines (77, 100). Furthermore, to enhance T cell function, SLAMF6 activates the small GTPase Rap1, thereby increasing T cell adhesiveness (113). In the cancer context, a study found that there is no difference at the level of phosphorylated ribosomal protein S6 regulating signaling pathways, including PI3K/AKT/mTOR and RAS-ERK between control T cells and SLAMF6^-/-^ T cells (114), suggesting SLAMF6 might be associated with these signaling pathways. Overall, the exact mechanism of SLAMF6 in cancer cell signaling remains to be elucidated.

### 2.6 SLAMF7

SLAMF7 is primarily expressed by NK cells, T lymphocytes, activated B cells, and macrophages (**Table 1**). One ITSM is found in the cytoplasmic domain of SLAMF7 and is involved in the interaction with SAP. The homophilic association between SLAMF7 and NK cells governs their cytolytic activity (65).

In cells lacking EAT-2 expression, following the homotypic interaction SLAMF7 between immune cells and myeloma cells, SHIP-1 recruitment is occurred, which requires tyrosine residue (Y-284) in the ITSM motif of SLAMF7. Thus, the immune cell is inhibited. In contrast, in cells with EAT-2 expression, following the interaction EAT-2 is mostly recruited to phosphorylated tyrosine residue (pY-304) within the ITSM of SLAMF7, and then PLCγ-1 and PLCγ-2 are recruited to phosphorylated tyrosine (pY-127) on EAT-2, which eventually leads to activate the ERK signaling pathway in immune cells. However, due to the absence of EAT-2 and CD45 expression in myeloma cells, these signaling pathways are revoked (115–117).

### 2.7 SLAMF8

SLAMF8 (CD353 or BLAME; B lymphocyte activator macrophage expressed) includes an extracellular and transmembrane domain. However, unlike most other SLAM members, it contains no ITSM motifs, resulting in the prevention of SAP-dependent function (69). SLAMF8 is found in monocytes, DCs, and neutrophils (**Table 1**). It plays a critical role in regulating inflammation by acting as a negative regulator of forming reactive oxygen species (ROS) and migration (70). A study has demonstrated that T cells and NK cells express SLAMF8, which is weakly expressed in B cells. In contrast, SLAMF8 expression was restricted in plasmablasts and plasma cells (71).

### 2.8 SLAMF9

SLAMF9 (also known as CD2F10) is found on myeloid cells (72), plasmacytoid DCs (73), tumor-associated macrophages (74), peritoneal B1 cells (75), and peritoneal macrophages (**Table 1**) (75).

## 3 SLAM molecules have a role in tumor immunity and cancer immunotherapy

### 3.1 SLAMF1

Several studies have expanded current knowledge on SLAMF1 in the pathophysiology of CLL and lymphoma (12, 85, 87, 118–120). A study has demonstrated that SLAMF1 acts as a negative regulator of IL-10 expression and secretion on the surface of B cell CLL and is associated with favorable clinical outcomes (12). Another study revealed that through selective suppression of Akt and MAPK signaling, the interaction of SLAMF1 and CD180 receptor pathways contributes to the pathobiology of B cell CLL (87). In Epstein-Barr virus (EBV)-positive B cell lymphoma cell lines, SLAMF1 has been associated with drug resistance and promoted cancer cell survival (121, 122). In contrast, a study reported no association between SLAMF1 levels and chemosensitivity in B cell CLL (123). However, in B cell CLL, a high level of mRNA expression of the mSLAMF1 isoform is related to fludarabine sensitivity and cyclophosphamide resistance. In contrast, a high level of nSLAMF1 mRNA expression indicates fludarabine resistance (123).

SLAMF1 and CD180 are considered positive CD20 expression regulators that may promote the responsiveness of SLAMF1^+^CD180^+^ B cell CLL to cancer immunotherapies based on CD20 targeted therapy (123).

It has been demonstrated that the up-regulation of SLAMF1 and SLAMF7 is a favorable prognostic marker. Patients with CLL that do not have SLAMF1 and SLAMF7 down-regulation have more effective NK cell-mediated killing and, as a result, may have improved immunological control (124). Consistent with these results, an investigation have reported down-regulation of SLAMF1 as an unfavorable prognostic marker on time-to-first treatment and overall survival (125). An empirical investigation indicated that loss of SLAMF1 expression in CLL alters genetic pathways that orchestrate chemotaxis and autophagy and treatment responses, raising the possibility that these alterations are responsible for the worse clinical outcomes observed by patients with SLAMF1 low expression (11). A research has indicated that B lymphoma cells susceptibility to oncolytic virotherapy with measles increases with the up-regulation of SLAMF1 expression (126).

In choriocarcinoma cell lines, SLAMF1 may increase methotrexate resistance by triggering protective autophagy (127). A paper has reported that the nSLAMF1 isoform is the main SLAMF1 isoform in glioma cells and suggested that SLAMF1 expression in the central nervous system (CNS) tumors can serve as a new diagnostic marker as well as a potential target for innovative therapeutic approaches in the future (128). An *in silico* study based on The Cancer Genome Atlas (TCGA) database reported that the three-gene signature, including SLAMF1/CD27/SELL, could be used as a biomarker to predict the prognosis and personalized immunotherapy for cervical cancer (129). A study reveals no expression of SLAMF1 on the cell surface membrane in breast cancer cell lines, while the prostate cancer cell lines expressed SLAMF1 both in the cytoplasm and on the cell surface (130).

### 3.2 SLAMF2

A therapeutic approach against lymphoid leukemia, lymphoma, and MM is a mAb directed against SLAMF2 because SLAMF2 is expressed in these malignancies (13)*. In vitro*, a developed in-house anti-SLAMF2 mAb elicits modest antibody-dependent cell-mediated cytotoxicity and significant complement-dependent cytotoxicity against MM cell lines and primary MM plasma cells. In mouse models injected subcutaneously with MM cells, administration of the anti-SLAMF2 mAb dramatically slows tumor development. It hinders the development of MM cells injected directly into mice’s bone marrow (BM). Notably, the anti-SLAMF2 mAb does not impair normal CD34^+^ hematopoietic stem cells (13). In patients with AML, SLAMF2 is a favorable prognostic marker that is down-regulated. AML may evade NK cell immunosurveillance by down-regulation of SLAMF2 expression (131). Methylation regulates SLAMF2 expression, and a hypomethylating drug may up-regulate SLAMF2 expression, increasing NK cell-mediated cytotoxicity *in vitro*. Furthermore, increased SLAMF2 expression *in vivo* may reverse AML immune evasion and boost NK cell activity (132). Using the NK-92MI cell line and SLAMF2-knockdown leukemia cells, an analysis asserted that NK-92MI-targeted knockdown cells show a phenotype of reduced NK susceptibility (133).

In patients with renal cell carcinoma (RCC), overexpression of SLAMF2, CD85, CD45, and PD-1 characterizes a predominantly inhibitory phenotype in circulating and tumor-infiltrating NK cells (134). In the HCC tumor microenvironment, growth differentiation factor 15 (GDF15) leads to anti-tumor immunosuppression *via* SLAMF2 expression on regulatory T cells (135). A study has concluded that SLAMF2 is a promising immunotherapeutic target in colorectal cancer (136).

### 3.3 SLAMF3

**Figure 3:**
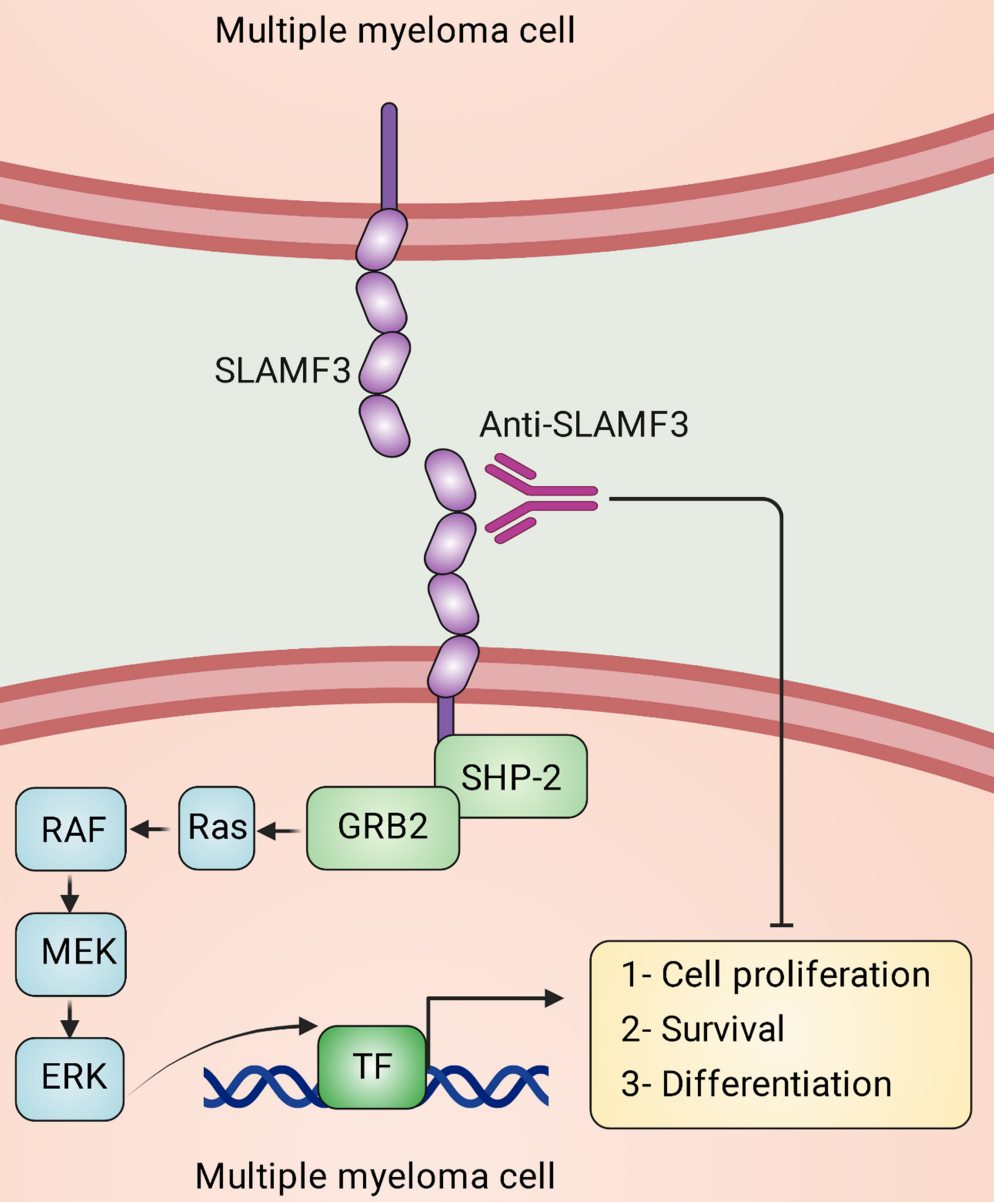
Signaling lymphocytic activation molecule family receptor (SLAMF) 3 signal transduction in multiple myeloma (MM) cells. In MM, homotypic interactions between SLAMF3 molecules are established, stimulating the ERK signaling pathway and specific transcription factors (TFs). This pathway eventually causes survival, proliferation, migration, differentiation, and anti-apoptotic responses in MM cells, which facilitates the progression of the malignancy. Anti-SLAMF3 antibodies might restrict the development of malignancy.

Several studies indicate up-regulated expression of SLAMF3 on MM cells (**Figure 3**) (101, 137–139). It has been found that SLAMF3 is strongly and constitutively expressed in MM cells independent of the malignancy stage and that SLAMF3 knockdown/knockout reduces proliferative capacity and promotes drug-induced apoptosis in MM cells (101). In addition, high levels of serum-soluble SLAMF3 may be a valuable prognostic indicator for MM development (101). It has been suggested that immunotherapies targeting SLAMF3 eradicate the majority of tumor cells (**Figure 3**) and aid in eliminating chemotherapy-resistant cells that remain in the BM following therapy in patients with MM (139).

Hepatocyte proliferation and hepatocellular tumorigenesis are both controlled by SLAMF3. In human HCC samples and cell lines, SLAMF3 expression is considerably lower than in healthy primary human hepatocytes. High levels of SLAMF3 expression are restored in HCC cell lines, inhibiting cell proliferation, and migration while increasing apoptosis (15). A study has reported that SLAMF3 down-regulates multidrugs resistance protein-1 (MRP-1) expression, causing HCC cells sensitization to chemotherapy drugs (140). SLAMF3 also reduces STIM1 expression, which eventually decreases breast cancer cell migration. This molecule might be used to predict breast cancer development and aggressiveness (141). SLAMF3 is identified as inhibitory immune checkpoint and “don’t eat me” receptor on macrophages, causing the inhibition of hematopoietic tumor cells phagocytosis mediated by macrophages (4). Considering that SLAMF3 is a B and T lymphocyte cell surface receptor that is robustly expressed on MM cells, Radhakrishnan and colleagues have generated SLAMF3 chimeric antigen receptor (CAR) T cells that are highly active against MM plasma cells, memory B cells, and MM-propagating cells *in vitro* and *in vivo* (142).

### 3.4 SLAMF4

In cancer studies, SLAMF4 plays a role in NK cells as an activating receptor and inhibitory receptor (**Figure 2**), CD8^+^ T cell exhaustion, and myeloid cells such as MDSCs (97). Additionally, SLAMF4 is identified as an inhibitory immune checkpoint and “don’t eat me” receptor on macrophages, causing the inhibition of tumor cells phagocytosis mediated by macrophages (4). Furthermore, SLAMF4 deletion improves phagocytosis of CD19-positive hematopoietic targets mediated by anti-CD19 CAR macrophages (4).

NK cells are essential for tumor cell surveillance. SLAMF4 is found in all NK cells, and it was in these cells that the signaling mechanisms of SLAMF4 were first discovered (97). Anti-SLAMF4 mAb therapy of mouse NK cells results in increased interferon-gamma (IFN-γ) production and non-major histocompatibility complex (MHC)-restricted tumor cell killing *in vitro* (46). Interactions between SLAMF4 and SLAMF2 are essential for optimum human NK cell proliferation in response to IL-2 and contribute to mouse NK cell proliferation, lytic capacity, and cytokine release (143, 144). Moreover, using anti-SLAMF4 mAb to crosslink SLAMF4 on human NK cells results in target cell lysis by NK cells (47). However, anti-SLAMF4 therapy inhibits IL2-stimulated proliferation in cultured human NK cells, showing that SLAMF4 might mediate activating or inhibitory signaling (47). Inlining with the inhibitory function of SLAMF4, murine NK cell effector activity is inhibited by SLAMF2^+^ target cells, inhibiting the SLAMF4-SLAMF2 interaction with specific mAb reverses this inhibition, resulting in increased target cell lysis (145). *In vitro*, SLAMF4 ligation reduces NK cell-mediated lysis of SLAMF2^+^ tumor cells and IFN-γ production by NK cells (48). *In vivo*, following anti-SLAMF4 mAb administration intravenously, the number of B16F10 syngeneic melanoma lung nodules in wild-type mice is considerably reduced (18). Tumor necrosis factor-alpha (TNF-α) and IFN-γ production are promoted when SLAMF4^+^ NK cells were co-cultured with tumor-infiltrating SLAMF2^+^/CD68^+^ monocytes/macrophages from patients with HCC. However, cytokine production and apoptosis decreases and increases, respectively (146). These findings establish an inhibitory role for SLAMF4 in mouse and human NK cells.

Tumor-infiltrating CD8^+^ T cells from HNSCC patients and mouse HNSCC-bearing models have significantly higher SLAMF4 expression linked with PD-1 molecule expression than healthy tissues. Additionally, SLAMF4 expression is higher on intratumoral DC and MDSC, and it is linked to PD-L1 expression and spontaneous production of immune-suppressive molecules. *In vitro*, SLAMF4 activation reduces the production of pro-inflammatory cytokines in human DCs. Notably, SLAMF4 knocked-out animal model dramatically reduces HNSCC tumor development. Interestingly, anti-SLAMF4 mAb therapy of mouse models reduces the growth of pre-existing HNSCC tumors and enhances tumor-infiltrating CD8^+^ T cells (16). SLAMF4 shows increased expression on exhausted CD8^+^ T cells in human cancers such as melanoma (147), MM (148), and AML (14). SLAMF4 is expressed on CD8^+^ T cells with an exhausted state in cancer animal models. T cells expressing co-inhibitory receptors SLAMF4, PD-1, and B- and T-lymphocyte attenuator (BTLA) are found in higher numbers in tumor-bearing animals compared to naive controls in syngeneic C57BL/6 mouse models of pancreatic adenocarcinoma and lung carcinoma (17).

SLAMF4 expression on MDSCs in tumor-bearing mice has been documented. A study found granulocytic myeloid-derived suppressor cells (Gr-MDSCs) expressing SLAMF4 in syngeneic tumor models. Additionally, significant variations in terms of function have been reported between SLAMF4^+^ Gr-MDSCs and SLAMF4^–^ Gr-MDSCs. SLAMF4^+^ Gr-MDSCs inhibit antigen-specific CD8^+^ T cell responses substantially more than SLAMF4^-^ Gr-MDSCs. Furthermore, SLAMF4 expression on Gr-MDSCs is linked to the generation of ROS and myeloperoxidase (149). Moreover, increased IL-2 and reduced IFN-γ production are linked to increased SLAMF4 expression on antigen-specific CD8^+^ T cells from the spleens of tumor-bearing mice (149).

### 3.5 SLAMF5

A study has recently investigated that SLAMF5 is expressed on MM cells, candidating a novel therapeutic target in MM (132). This study indicates that cells producing macrophage migration inhibitory factor (MIF) induce SLAMF5 expression on cells in MM microenvironment and result in MDSCs accumulation and up-regulated PD-L1 expression on MDSCs (150). SLAMF5 inhibition diminishes MDSCs accumulation, increasing T cell activation and decreasing tumor burden (150). SLAMF5 acts as a regulator in favor of malignancy in CLL (109, 151–153). It has been suggested targeting SLAMF5 on BM-derived DCs as a novel therapeutic strategy, which may reduce CLL (151). SLAMF5 on CLL cells interacts with SLAMF5 on microenvironment cells, promoting cell survival on both sides*. In vitro* and *in vivo*, SLAMF5 inhibition causes CLL cell death (109). An investigation found that SLAMF5 promotes PD-L1 expression in CLL cells and their microenvironment and PD-1 expression in T cells (152).

### 3.6 SLAMF6

SLAMF6/Ly108 facilitates macrophage M2 polarization, which aids in developing hepatocellular cancer (154). mAb directed against SLAMF6 considerably reduces the peritoneal cavity leukemic load and slows tumor development in B cell-related leukemias, lymphomas, and nonhematopoietic cancers such as melanoma. Moreover, anti-human SLAMF6 mAb restores exhausted CD8^+^ T cells *in vitro* (111).

According to the findings of a study, SLAMF6 is an inhibitory immune checkpoint whose absence allows potent CD8^+^ T cells to eliminate melanoma cells (114). TCR triggering of anti-melanoma CD8^+^ T cells in SLAMF6 knockout mice resulted in a robust effector phenotype, increased IFN-γ production, enhanced cytolysis, and improved outcomes in the adoptive transfer of SLAMF6^-/-^ anti-melanoma CD8^+^ T cells to treat existing melanoma (114).

### 3.7 SLAMF7

**Figure 4:**
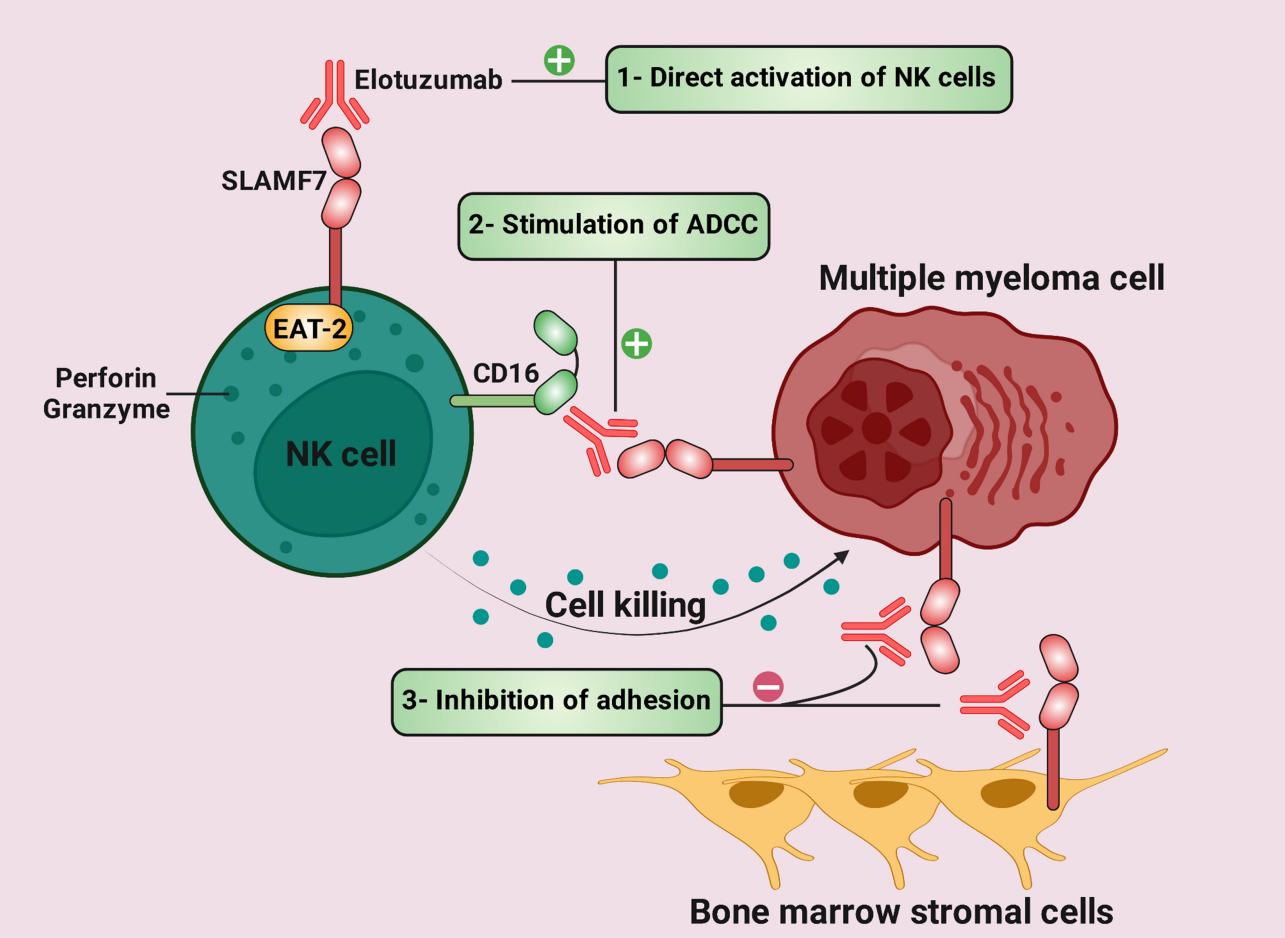
Therapeutic mechanisms of elotuzumab in multiple myeloma (MM) (1). Elotuzumab can bind to signaling lymphocytic activation molecule family receptor (SLAMF) 7, thus stimulating direct activation of natural killer (NK) cells *via* EAT-2. **(2)** While the Fc component of elotuzumab interacts with the activating Fc receptor CD16 on NK cells, the Fab portion binds to SLAMF7 on MM cells. This interaction results in antibody-dependent cellular cytotoxicity (ADCC). **(3)** Elotuzumab prevents MM cells from adhering to bone marrow stromal cells (BMSCs) because BMSCs and MM cells interact via SLAMF7-SLAMF7 homotypic interactions.

SLAMF7 is up-regulated by about 97% of myeloma cells, although its expression in normal cells is limited (155, 156). SLAMF7 was targeted in preclinical investigations by HuLuc63 (Elotuzumab; Elo) (155). HuLuc63 prevents MM cells from attaching to stromal cells in the BM and causes antibody-dependent cellular cytotoxicity (ADCC) against MM cells (**Figure 4**) (155, 157). Elo enhances ligation between NK and myeloma cells and induces NK cytotoxicity against myeloma cells in an ADCC-independent manner (158, 159). Moreover, Elo improves NK cell cytotoxicity against MM cells in a CD16-independent manner and promotes SLAMF7-SLAMF7 interactions (**Figure 4**) (160).

Elo can potentially activate ADCC in MM cells resistant to bortezomib (a proteasome inhibitor) and HSP90 inhibitors (6). A study found that combining Elo and bortezomib significantly exhibits *in vivo* anti-tumor activity in the MM xenograft model and enhances Elo-induced MM cell lysis (156). In phase 1 and the dose-escalation trial, Elo was generally well tolerated in patients with advanced MM, while it had no same efficiency as in the animal model (161). Elo increases the objective response rate (ORR) from 45% to 48% when given to patients on bortezomib therapy (162). In the phase II clinical trial, the combination of Elo, lenalidomide, and dexamethasone in 150 patients with MM improved progression-free survival from 6.9 to 9.7 months with a steady ORR from 66 to 63% (163). Elo has been shown to enhance ORR by 82% in combination with lenalidomide and low-dose dexamethasone (164). **Table 2** provides more clinical trials regarding Elo therapy in patients with cancer.

**Table 2:** Agents targeting SLAM-family receptors under preclinical and clinical investigations.

Target	Type of cancer	Type of treatment	Agent	Phase	References
SLAMF1	ALL	Oncolytic virotherapy with measles virus	CAM-70	PC	(126)
	NHL	Oncolytic virotherapy with vaccine-identical measles virus	MV^vac2^NIS	PC	(165)
SLAMF2	B cell lymphoma	mAb therapy	HuLy-m3	PC	(166)
	MM	mAb therapy	1B4	PC	(13)
	CLL	mAb therapy	WM63	Phase 1 clinical trial	(167)
	MM	Antibody-drug conjugate	SGN-CD48A	Phase 1 clinical trial (terminated)	(168)
SLAMF3	Hematopoietic cancer	CAR macrophage therapy+SLAMF3 deletion	Anti-CD19 CAR macrophage	PC	(4)
	MM	CAR T cell therapy	Anti-SLAMF3 CAR T cell	PC	(142)
SLAMF4	Leukemia, Neuroblastoma	Chimeric receptor	2B4-ζ	PC	(169)
	Hematopoietic cancer	CAR macrophage therapy+SLAMF4 deletion	Anti-CD19 CAR macrophage	PC	(4)
SLAMF5	MM	mAb therapy	B4	PC	(150)
SLAMF6	Lymphoma, CLL	mAb therapy	994.1, 480.12	PC	(170)
	CLL	mAb therapy+ Bruton tyrosine kinase inhibitor	αSLAMF6+Ibrutinib	PC	(171)
SLAMF7	MM	SLAMF7-specific immunogenic peptide	SLAMF7_239-247_	PC	(172)
		CAR T cell therapy+ Immunomodulation	Anti-SLAMF7 CAR T cell+ Lenalidomide	PC	(173)
		mAb therapy	HuLuc63 (Elotuzumab or BMS-901608)	PC	(155, 157)
		mAb therapy+ proteasome inhibitor	Elotuzumab+Bortezomib	PC	(156)
		mAb therapy+ Immunomodulation	Elotuzumab+Lenalidomide+Dexamethasone	Phase 3 clinical trial	(174)
	Relapsed/refractory MM	mAb therapy+ proteasome inhibitor	Elotuzumab+Bortezomib	Phase 1 clinical trial	(162)
		mAb therapy+ Immunomodulation	Elotuzumab+Lenalidomide+Dexamethasone	Phase 1 clinical trial	(164)
		mAb therapy+ Immunomodulation	Elotuzumab+Lenalidomide+Dexamethasone	Phase 3 clinical trial	(175)
		mAb therapy+ proteasome inhibitor+ Immunomodulation	Elotuzumab+Bortezomib +Dexamethasone	Phase 2 clinical trial	(163)
		mAb therapy+ Immunomodulation	Elotuzumab+Thalidomide+Dexamethasone	Phase 2 clinical trial	(176)
	MM with renal impairment	mAb therapy+ Immunomodulation	Elotuzumab+Lenalidomide+Dexamethasone	Phase 1b clinical trial	(177)
		mAb therapy	Elotuzumab	Phase 1 clinical trial	(161)
		mAb therapy+ Immunomodulation	Elotuzumab+Lenalidomide+Dexamethasone	Phase1b-2 clinical trial	(178)

ALL, Acute lymphocytic leukemia; CLL, Chronic lymphocytic leukemia; PC, Preclinical; MM, Multiple myeloma; mAb, Monoclonal antibody; CAR, Chimeric antigen receptor; SLAM, Signaling lymphocytic activation molecule.

Immunogenic peptides from the SLAMF7 antigen effectively activate specific cytotoxic T lymphocyte (CTL) clones. Accordingly, an immunogenic HLA-A2-specific SLAMF7_239-247_ peptide can induce antigen-specific CTL against MM cells, lysing tumor cells (172).

An empirical study highlighted that B16-F10 tumors grow more slowly in mice lacking SLAMF7 (179). Additionally, CD8^+^ T cells express lower levels of PD-1 and have a reduced capacity to manifest a T cell exhaustion phenotype in this animal model (179).

SLAMF7 is a decent target for CAR T cell therapy because of its high expression on myeloma cells and low expression on normal cells (173, 180–183). The distal variable (V) domain and the proximal constant 2 (C2) domain are two extracellular domains of the SLAMF7 protein. O’Neal and colleagues developed and evaluated SLAMF7 CAR T cells targeting the V domain of SLAMF7 (Luc90-SLAMF7 CAR T) and demonstrated anti-myeloma killing *in vitro* and *in vivo* using two myeloma mouse models (180). Another study corroborates the efficacy of SLAMF7 CAR T cells against MM cells. Adoptive transfer of SLAMF7 CAR T cells into MM-bearing mice shows potent anti-cancer efficacy. *In vitro*, adding lenalidomide to SLAMF7 CAR T cell improves immune functions such as cytotoxicity, memory maintenance, Th1 cytokine production, and immune synapse formation. Furthermore, the anti-cancer activity and durability of adoptively transferred SLAMF7 CAR T cells are also increased by lenalidomide *in vivo* (173).

One concern regarding SLAMF7 CAR T cell therapy may limit its use in the clinic. This limitation is that SLAMF7 is expressed in normal NK cells. Thus, incorporating a suicide gene with an anti-SLAMF7 CAR is reasonable. Accordingly, Amatya and colleagues invented a construct with a suicide gene and a CD28-containing anti-SLAMF7 CAR. The suicide gene encodes a dimerization domain linked to a caspase-9 domain. *In vitro*, T cells carrying the anti-SLAMF7 CAR with suicide-gene construct detect SLAMF7 and eradicate tumors in mice. Finally, the dimerizing chemical rimiducid is used to kill T cells that express this construct, preventing damage to normal immune cells (184).

### 3.8 SLAMF8

A study has revealed that anaplastic large-cell lymphoma cell lines express SLAMF8 (185). Furthermore, SLAMF8 knockdown down-regulates SHP-2 activation and cell proliferation while increasing apoptosis in lymphoma cell lines (185). SLAMF8 orchestrates oncogenic KIT-mediated RAS-RAF-ERK signaling and SHP-2-mediated human neoplastic mast cell proliferation (186). Findings from analysis of the TCGA database revealed that high SLAMF8 expression is a prognostic factor in glioma, conferring reduced overall survival, chemotherapy resistance, and aggravated immunosuppression (187). In addition, high SLAMF8 expression is a favorable prognostic factor for better anti-PD1 immunotherapy efficacy in patients with gastrointestinal cancers (188).

### 3.9 SLAMF9

SLAMF9 expression was discovered in malignant cell lines from the monocytic and lymphocytic origin using RT-PCR (189). In macrophages, SLAMF9 is a novel type-I transmembrane receptor with immunomodulatory characteristics. SLAMF9 affects pro-inflammatory cytokine production and migration in murine and human melanoma tumor-associated macrophages (74).

## 4 Potential challenges ahead in targeting the SLAM family members emphasizing knowledge gaps

There are numerous potential hurdles in targeting SLAM family members, which deserve special attention. In the following, these challenges will be examined.

### 4.1 Potential toxicities and adverse effects (AEs)

As discussed earlier, targeting SLAM molecules has been explored as a potential therapeutic strategy in cancer. However, as with any therapeutic target, there are potential toxicities associated with targeting SLAM family members. Some of the toxicities and AEs observed in preclinical and clinical studies include lymphopenia, fatigue, chest pain, gastroenteritis, neutropenia, thrombocytopenia, and hepatotoxicity (162, 164, 175, 176, 178, 190, 191). These AEs might due to the broad expression of SLAM family members on various immune cells and their complex regulatory functions within the immune system. Furthermore, targeting SLAM family members might also affect the normal immune response and increase the risk of infections, as some of these receptors are involved in controlling immune cell function (90), including the clearance of pathogens (21, 192). Therefore, careful consideration should be given to the potential toxicities associated with targeting SLAM family members, and appropriate safety measures should be taken to minimize the risk of AEs.

### 4.2 Networking of SLAM molecules with other immune checkpoints

There is currently no clear evidence to suggest that targeting the SLAM receptors on cancer cells would lead to the up-regulation of other checkpoint molecules. The association between SLAM receptors and other checkpoint molecules such as PD-1, CTLA-4, and T cell immunoglobulin and mucin domain-containing protein 3 (TIM-3), is not well established. However, a study reported that SAP inhibits PD-1 signaling in T cells by interacting with CD28 (193). To prevent SHP-2 from dephosphorylating downstream phosphorylated CD28, SAP has indirect interactions with the PD-1 signaling complex (194). Additionally, in primary human T cells, suppressing SAP improves PD-1 ligation and function, which in turn suppresses IL-2 production (194, 195). Thus, it is postulated that targeting SLAM receptors could have downstream effects on other immune checkpoint molecules, but more research is needed to understand the potential interactions and outcomes of such therapies.

### 4.3 Dual function: inhibitory or stimulatory role

The function of SLAM receptors, including SLAMF1/3/4/6/7, is dichotomous (**Figure 1**). SLAMF3 function, for instance, is intricate and not well understood as it may have inhibitory (196) or stimulatory (197, 198) functions depending on the context of the immune response. SLAMF3 has been shown to have different effects on cancer depending on the cell types and the signaling pathways involved. In some cases, SLAMF3 has been reported to have an inhibitory effect on cancer. For instance, studies have shown that SLAMF3 can inhibit the growth and proliferation of certain types of cancer cells such as liver cancer and hematopoietic tumors (4, 15, 140). However, in other cases, SLAMF3 has been found to have a stimulatory effect on cancer. For instance, SLAMF3 has been shown to induce the proliferation of MM cells (101).

SLAMF7 appears to have both inhibitory and stimulatory effects in cancer, depending on the type of cancer and context in which it is expressed. In some types of cancer, such as MM and breast cancer, SLAMF7 has been found to be overexpressed (199), and its presence on cancer cells might aid them evade attack by the immune system. In this context, immunotherapies targeting SLAMF7, such as Elo and CAR T cell therapy, have been developed (173, 180–183, 200). On the other hand, in some other types of cancer, such as ovarian cancer and RCC, low expression of SLAMF7 has been associated with poor prognosis (179), suggesting that SLAMF7 may play a role in suppressing tumor growth in these contexts. Therefore, the impact of SLAMF3 and SLAMF7 on cancer is context-dependent and requires further investigation.

Overall, further study is required to identify if a given SLAM receptor acts as an activatory receptor or an inhibitory receptor in each cancer type. Functional assays using cancer cells and SLAM receptor-specific antibodies or CAR T cells might be one such strategy. This would aid in determining whether or not SLAM receptor activation enhances the development and survival of cancer cells. Instead, genetic knockdown or knockout approaches might be used to examine the impact of SLAM receptor deletion on the behavior of tumor cells. Eventually, investigating SLAM receptor expression in patient samples might help identify biomarkers and therapeutic targets for various cancers.

### 4.4 On-target off-tumor effect

In the case of targeting SLAM molecules, the potential on-target off-tumor effects could include immune system dysfunction, as SLAM molecules play a crucial role in immune cells function. Not all SLAM receptors are expressed only on normal immune cells. While these receptors have a widespread expression on different immune cells such as T cells, B cells, and NK cells, also these molecules may be expressed in malignant cells. For instance, SLAMF7 is expressed in MM cells (201). Hence, immunotherapeutic strategies targeting SLAMF7 should be deployed with caution in patients with MM because of SLAMF7 expression on non-cancerous immune cells. One approach to minimize the risk of on-target off-tumor toxicities and optimize the benefits of targeting SLAM molecules might involve combining SLAM-targeted therapy with immune checkpoint inhibitors, which could help to mitigate these AEs and enhance the efficacy of the treatment.

## 5 Conclusion and future directions

Our knowledge of the role of SLAM-family receptors in cancer immunity has grown during the last several years. There is growing irrefutable proof that receptors belonging to the SLAM family mediate essential functions in immune cells. Studies have indicated that the SAP family of adaptors decides whether a SLAM receptor has a stimulatory or inhibitory function. Additionally, findings have established that SLAMF7 is a valid and safe drug target for the treatment of MM, and results statistically showed significant trends.

This article presented an outline of SLAM molecules with a focus on recent and previous findings on the role of SLAM family molecules in treating various cancers. Evidence indicates that some members of this family act as an inhibitory immune checkpoint. Thus, these molecules represent a novel therapeutic avenue to boost antitumoural immunity. Also, these molecules may be used as a predictor of cancer development and aggressiveness. Given recent breakthroughs in current knowledge and our understanding of the role of the SLAM-family receptors in tumor immunity, the receptors within this axis are potential candidates for hematological and solid tumors immunotherapy. However, a significant hurdle limiting the translation of ICIs to clinical therapeutic approaches is the corresponding AEs. For instance, serious AEs were reported in most patients with cancer receiving Elo (174). Overall, we tried to provide some powerful arguments which will shape our thinking about SLAM molecules in the context of cancer for the next years.

Several unresolved questions remain to be answered. 1 – Is the expression of SLAM molecules associated with the tumor stage? 2 – Which biomarkers predict the patient’s therapeutic response to SLAM inhibition? 3 – Considering the alterations in the elderly patients’ immune system, is the therapeutic response of this patient population to the inhibition of the SLAM molecule similar to other patients? 4 – Is there a possibility of any immune-related AEs associated with SLAM inhibition?

Several emerging therapies have been used in association with SLAM molecules. One of these novel paradigms is CAR T cells directed against SLAMF3 and SLAMF7. Clinical trials assessing the effects of these CAR T cells on progression-free survival and overall survival might be an intriguing research direction in solid and liquid tumors. mAb directed against SLAMF7, such as HuLuc63 or Elo, has emerged as a powerful tool in the armamentarium against MM (**Figure 4**). Many studies assessed the efficacy of Elo alone or in combination with therapeutic agents in MM. However, this mAb has not been applied in solid tumors and remains a conspicuous area of research with potential relevance to cancer patients. One of the main criticisms of targeting SLAM molecules is that they are also expressed in normal immune cells. This point limits the bench-to-bedside clinical success of SLAM-based targeted immunotherapies. Thus, preclinical and clinical investigations should examine whether SLAM molecule inhibitors have significant AEs. The definitive goal in combating tumors is to achieve the specific recognition and effective eradication of tumor cells with minimum and tolerable AEs. A significant hurdle limiting bench-to-bedside translation of ICIs directed against SLAM molecules is potential AEs. Overall, anti-SLAM mAbs-related toxicity might be a rare but severe side effect that deserves special attention. In order to effectively manage cancer patients who have toxicity due to SLAM-targeted immunotherapies, a better knowledge of the cause, early identification, and timely treatment are vital. Thus, further investigations underlying anti-SLAM mAbs-associated toxicity need to be undertaken.

## Author contributions

PF and A-AD conceptualized the study. PF, ShamM, ShabM, and HN drafted the manuscript. MA and A-AD revised the article critically for important intellectual content. MA and A-AD supervised the study. All authors contributed to the article and approved the submitted version.
